# 

**Published:** 1841-07

**Authors:** 


### CONTENTS OF No. XXIII.

OF THE

### British and Foreign Medical Review.

JULY, 1841.

---

### PART FIRST.—Analytical and Critical Reviews.

PAGE

ART. I.—Principes généraux de Statistique Médicale, ou Développement des Règles qui doivent présider à son emploi. Par JULES GAVARRET . . 1
General Principles of Medical Statistics, or a Development of the Rules which ought to preside over the use of Numbers in Medical Investigations. By JULES GAVARRET.

ART. II.—Recherches Anatomiques, Pathologiques et Thérapeutiques, sur la Maladie connue sous les noms de Fièvre Typhoïde, Putride, Adynamique, Ataxique, Bilieuse, Muqueuse, Gastro-entérite, Entérite Folliculeuse, Dothinentérie, &c. comparée avec les Maladies aigues les plus ordinaires. Par P. C. A. LOUIS . . . . . . . . 22
Anatomical, Pathological, and Therapeutical Researches on the Disease known by the names of Typhoid Fever, &c. By P. C. A. LOUIS.

ART. III.—Observations on the Religious Delusions of Insane Persons, and on the Practicability, Safety, and Expediency of imparting to them Christian Instruction; with which are combined a copious practical Description and Illustration of all the principal Varieties of Mental Disease, and its appropriate Medical and Moral Treatment. By NATHANIEL BINGHAM . . 54

ART. IV.—A Treatise on the Nervous Diseases of Women; comprising an Enquiry into the Nature, Causes, and Treatment of Spinal and Hysterical Affections. By THOMAS LAYCOCK, M.D. . . . . . . 60

ART. V.—1. Traité Complet, Théorique et Pratique, des Maladies du Foie. Par AUG. BONNET . . . . . . . 78
Complete Theoretical and Practical Treatise on Diseases of the Liver. By AUG. BONNET, M.D.

2. A Practical Treatise on the Diseases of the Liver and Biliary Passages. By WILLIAM THOMSON, M.D. . . . . . . ib.

3. The Madras Quarterly Medical Journal. Edited by SAMUEL ROGERS . ib.

ART. VI.—The Library of Medicine. Arranged and edited by ALEXANDER TWEEDIE, M.D. F.R.S., &c. . . . . . . 92

ART. VII.—A Letter to Sir Benjamin Brodie, F.R.S., &c., containing a critical enquiry into his 'Lectures illustrative of certain Local Nervous Affections.' By WILLIAM GOODLAD, M.R.C.S., &c. . . . . . 121

ART. VIII.—Researches, Physiological and Anatomical. By JOHN DAVY, M.D. F.R.S. 129

ART. IX.—The Anatomy of Suicide. By FORBES WINSLOW . . 149

ART. X.—Die Organischen Knochen-Krankheiten. Ein Lehrbuch von Dr. A. L. RICHTER . . . . . . . . 154
The Organic Diseases of the Bones. A Manual by Dr. A. L. RICHTER.

ART. XI.—The Sanative Influence of Climate; with an account of the best places of resort for Invalids in England, the South of Europe, &c. By Sir JAMES CLARK, Bart., M.D. F.R.S. . . . . . . 160

ART. XII.—De la Nécessité des Etudes Pratiques en Médecine Légale, &c. Par H. L. BAYARD . . . . . . . 174
On the Necessity of Practical Instruction in Medical Jurisprudence. By Dr. BAYARD.

ART. XIII.—1. Practical Observations on Distortions of the Spine, Chest, and Limbs; together with remarks on Paralytic and other Diseases connected with Impaired or Defective Motion. By W. T. WARD, F.L.S., &c. . 177

2. Spinal Curvature, its Consequences and its Cure; illustrated by the History of Thirty-three Cases, successfully treated. By J. B. SERNY, M.D., &c. . ib.

§

4289

PAGE

3. Practical Remarks on the Causes, Nature, and Treatment of Deformities of the Spine, Chest, and Limbs, Muscular Weakness, Weak Joints, Muscular Contractions, and Stiff Joints, containing the results of the author's experience, and showing the advantages derived from the modes of treatment which he has recently introduced. With illustrative Plates and Cases. By J. Amesbury 177
4. On a new Operation for the Cure of Lateral Curvature of the Spine, with Remarks on the Causes and Nature of that Disease. By Fred. C. Skey, F.R.S. ib.
5. Spinal Diseases; with an improved Plan of Cure, including what are commonly called Nervous Complaints, and numerous examples from upwards of 150 Cases. By John Henry Robertson, M.D., &c. . . . ib.
6. The Cause and Treatment of Curvature of the Spine, and Diseases of the Vertebral column. By E. W. Tuson, F.R.S. F.L.S., &c. . . . ib.
7. Spinal Affections. A popular Lecture on Disorders and Diseases of the Spine. By H. C. Roods . . . . . . ib.
8. Série de Mémoires sur les Difformités du Système osseux. Avec Planches. Par le Dr. Jules Guerin . . . . . . ib.
Series of Memoirs on Deformities of the Bony System, &c. By J. Guerin, M.D. ib.

Art. XIV.—Wahres und Falsches in der sogenannten Wasserheilkunde. Von Dr. H. Claessen . . . . . . . 189

The Truth and Falsehood of the so-called Cold Water Cure. By Dr. H. Claessen.

Art. XV.—Mémoire sur l'Opération de la Taille. Par M. Souberbielle . 194

Memoir on the Operation of Lithotomy. By M. Souberbielle.

Art. XVI.—On the Diseases and Derangements of the Nervous System. By Marshall Hall, M.D. F.R.S. . . . . . . 200

Art. XVII.—1. Memoir on the Radical Cure of Stuttering by a Surgical Operation. By J. F. Dieffenbach . . . . . . 209

2. Du Bégaiement, &c. Par le Dr. C. Phillips . . . . ib.

On Stammering, &c. By Dr. C. Phillips.

3. Stammering and other Imperfections of Speech treated by Surgical Operations on the Throat. By James Yearsley, M.R.C.S. . . . ib.

4. On Stammering and Squinting, and on the Methods for their removal. By Edwin Lee, M.R.C.S. . . . . . . ib.

Art. XVIII.—Essai sur un Traitement méthodique de quelques Maladies de la Matrice. Injections intra-vaginales et intra-utérines. Par Aug. Vidal, &c. 215
Essay upon the methodical Treatment of certain Diseases of the Womb by Vaginal and Uterine Injections. By Aug. Vidal, &c.

---

### Part Second.—Bibliographical Notices.

Art. I.—The Physiology of Vision. By William Mackenzie, M.D. . . 217

Art. II.—A General Outline of the Animal Kingdom, and Manual of Comparative Anatomy. By Thomas Rymer Jones, F.L.S. . . . 218

Art. III.—Transactions of the Entomological Society of London . . 219

Art. IV.—The Prescriber's Pharmacopœia: containing all the Medicines in the London Pharmacopœia arranged in classes according to their action, with their composition and doses. By a Practising Physician . . 221

Art. V.—The American Medical Almanac for 1841. By J. V. C. Smith, M.D. . 223

Art. VI.—A Winter in the Azores, and a Summer at the Baths of the Furnas. By Joseph Bullar, M.D., and Henry Bullar . . . 224

Art. VII.—Bemerkungen über die Weise wie die Oeffnung in dem Schädel nach der Trepanation, oder anderem Knochenverlust, ausgefüllt wird. Von Dr. G. Vrolik . . . . . . . 226

Remarks on the manner in which the Opening in the Skull caused by the Trephine, or other loss of Bone, is filled up. By Dr. G. Vrolik.

Art. VIII.—Popular Cyclopædia of Natural Science—Vegetable Physiology. Published by the Society for the Promotion of Popular Instruction . . 227

Art. IX.—A Treatise on the Physiological and Moral Management of Infancy. By Andrew Combe, M.D., &c. . . . . . 228

Art. X.—Traité sur l'Hydrocephale Interne. Par G. Vrolik . . ib.

Treatise on Internal Hydrocephalus. By G. Vrolik.

PAGE

Art. XI.—Ueber Tuberkulose als die gewohnlichste Ursache des Hydrocephalus Auctus. Von Dr. F. Schweninger . . . . . 229

On Tubercular Degeneration as the most frequent cause of acute Hydrocephalus. By Dr. Schweninger.

Art. XII.—The Transactions of the Provincial Medical and Surgical Association 231

Art. XIII.—Rambles in Europe in 1839, with Sketches of Prominent Surgeons, Physicians, Medical Schools, Hospitals, Literary Personages, &c. By William Gibson, m.d. . . . . . . 232

Art. XIV.—Medizinische Zustände und Forchungen im Reiche der Krankheiten. Von Dr. Robert Volz . . . . . . 234

Medical Facts and Observations upon Diseases. By Dr. Robert Volz.

---

### Part Third.—Selections from the British and Foreign Journals.

### I. THE FOREIGN JOURNALS.

#### ANATOMY AND PHYSIOLOGY.

Ed. Engelhardt on the Functions of the Upper and Lower Halves of theSpinal Cord 235
A. W. Volkmann on the Anastomoses of Nerves . . . . . 236
M. Longet on the Functions of the Roots of the Nerves . . . . 239
A. W. Volkmann on the Motor Influences of the Cerebral and Cervical Nerves . ib.
Professor Bischoff on Electrical Currents in the Nerves . . . . 245
Dr. Erdl on the Circulation in the Infusoria . . . . . ib.
Dr. Kuerschner on the Functions of the Columns of the Spinal Cord . . ib.

#### PATHOLOGY, PRACTICAL MEDICINE, AND THERAPEUTICS.

Dr. Grahl on Croup and its Cure . . . . . . . . 246
Dr. Brunzlow on communication of Intermittent Fever from a Mother to her Child ib.
Grandoni's Poisoning of Leeches by the Blood of the Sick . . . 247
M. Aran on the Treatment of Acute Articular Rheumatism by Nitrate of Potash . ib.
Dr. Bidder on the Pathology of Plica Polonica . . . . . . ib.
Dr. F. A. v. Ammon on Horny Excrescences on the Eyelids . . . 248
Poisoning by Solution of Acetate of Lead.—Lead found in the Urine . . 249
Antimony in the Urine . . . . . . . . . . . ib.
M. Trousseau on the Employment of Arsenious Acid in Phthisis Pulmonalis . ib.
Dr. Karawagen on Scorbutic Pericarditis and Pleuritis, and the Cure by Paracentesis 250
Dr. Aubert on certain Anthelmintics in use in Abyssinia . . . ib.

#### SURGERY.

M. Reybard on preventing the Entrance of Air into the Chest in the Operation for Empyema . . . . . . . . 251
M. Lucien Boyer's Section of the Tendons of the Muscles of the Eye in the Horse ib.
M. Ricord on Tincture of Iodine as a topical Application to Phagedenic Chancres ib.
Mr. Guersent, Jun. on Hemorrhage of the Genio-glossi Muscles for Stammering . 252
M. Rigal's New System of Bandaging . . . . . . . ib.
M. Bérard on Ectropion Cured by Autoplasty . . . . . ib.
M. Roux on Excision of the Elbow-joint . . . . . . . 253
M. Bouvier on Dividing the Muscles of the Back in Lateral Curvatures of the Spine ib.
M. Bouley's Case of Intestinal Invagination in the Cow, cured by Gastrotomy . ib.
Dr. Kuh on Improvement of Defects in the Refractive Power of the Eye, by Myotomy ib.
M. S. de Renzi's Lateral Operations for Stone in Naples, with Statistics of Lithotomy 254
Dr. Hauck on the present mode of Treating the Itch at Berlin . . . ib.
M. Jobert's Cure of an ununited Fracture of the Humerus by a Seton . . 255
M. Dieulafoy's New mode of Treating Orchitis . . . . . . 256
M. Pescheux's Remarkable Case of Bronchotomy from a Bean in the Air-passages ib.
Dr. F. Rizzoli's Removal of a large part of the Rectum affected with Scirrhus . ib.
Notice respecting the Operation for Stammering . . . . . . 257

PAGE
Dr. P. Doubovitski on the subcutaneous Division of the Pronator and other Muscles 257
Drs. Woppisch and Bauer on the Treatment of Pseudarthrosis . . . . ib.
Dr. Lohsse's Iodine in Opacity of the Cornea . . . . . . 258
M. Bonnet's Anatomy of the Aponeuroses and Muscles of the Eye, in Strabismus ib.
M. Bouvier on Congenital Dislocation of the Femur . . . . 259
MM. Trousseau and Contour on Compression in Mammary Abscess . . ib.
Dr. Guyon's Statistics of Amputation in the African Army, in Hospitals and Field 260
M. Guyon's Operations of Laryngotomy . . . . . . . ib.

MIDWIFERY.

Dr. Hecking's Hemorrhage after Delivery arrested by a new Method . . 261
M. Ledesma's Hernia of the Uterus, and Cæsarean Operation . . . ib.
Dr. Alken's Retroversion of the Unimpregnated Uterus . . . 262
M. Maisonneuve's Extraction of a Foreign Body implanted in the Uterus . . ib.
M. Hourmann's Experiments on Uterine Injections . . . . 263
Pr. Von D'Outrepont on the Microscopic Characters of healthy Milk . ib.

MEDICAL JURISPRUDENCE AND TOXICOLOGY.

M. Devergie on Asphyxia from Carbonic Acid . . . . . 264
M. Orfila on the Signs of Suspension before and after Death . . . 270

CHEMISTRY.

Dr. Louis Mandl on the Chemical Analysis of the Blood in its Morbid Conditions . 273

STATISTICS.

Kohl's Account of the Petersburg Foundling Hospital . . . . 273

II. THE AMERICAN AND COLONIAL JOURNALS.

PATHOLOGY.

Flint's Remarks on Dyspepsia as connected with the Mind . . . 274

III. THE BRITISH JOURNALS.

ANATOMY AND PHYSIOLOGY.

Dr. Reid on the Relation between Muscular Contractility and the Nervous System 277
Dr. Reid on the Order of Succession in which Vital Actions are arrested in Asphyxia 278

PATHOLOGY, PRACTICAL MEDICINE, AND THERAPEUTICS.

Dr. Lowe's Jaundice from Non-Elimination, with Remarks on the Nature of Bile . 280
Dr. Griffin on the Diagnosis of Abdominal Inflammations . . . ib.
Mackenzie's Observations on Dropsy of the Pericardium . . . . 281
Leigh's new method of employing Iodine in Phthisis . . . . 282

SURGERY.

Nunneley on the Employment of Threads of Caoutchouc for Sutures . . 282
Lambert's Case of Neuralgia Facialis, cured by Operation . . . 283
Dr. Hunter on the Section of Muscles in Spinal Curvature . . . ib.
Southam on the use of Splints in Chorea . . . . . . 284

Part Fourth.—Medical Intelligence.

New Bills of Mortality for London . . . . . . . 285

Memoir of Dr. Hope . . . . . . . . . 286

Establishment of Hospitals in China . . . . . . . 290

Books received for Review . . . . . . . 291

